# SERCA2 Haploinsufficiency in a Mouse Model of Darier Disease Causes a Selective Predisposition to Heart Failure

**DOI:** 10.1155/2015/251598

**Published:** 2015-05-03

**Authors:** Vikram Prasad, John N. Lorenz, Valerie M. Lasko, Michelle L. Nieman, Wei Huang, Yigang Wang, David W. Wieczorek, Gary E. Shull

**Affiliations:** ^1^Department of Molecular Genetics, Biochemistry and Microbiology, University of Cincinnati College of Medicine, Cincinnati, OH 45267, USA; ^2^Department of Cellular and Molecular Physiology, University of Cincinnati College of Medicine, Cincinnati, OH 45267, USA; ^3^Department of Pathology and Laboratory Medicine, University of Cincinnati College of Medicine, Cincinnati, OH 45267, USA

## Abstract

Null mutations in one copy of *ATP2A2*, the gene encoding sarco/endoplasmic reticulum Ca^2+^-ATPase isoform 2 (SERCA2), cause Darier disease in humans, a skin condition involving keratinocytes. Cardiac function appears to be unimpaired in Darier disease patients, with no evidence that SERCA2 haploinsufficiency itself causes heart disease. However, SERCA2 deficiency is widely considered a contributing factor in heart failure. We therefore analyzed *Atp2a2* heterozygous mice to determine whether SERCA2 haploinsufficiency can exacerbate specific heart disease conditions. Despite reduced SERCA2a levels in heart, *Atp2a2* heterozygous mice resembled humans in exhibiting normal cardiac physiology. When subjected to hypothyroidism or crossed with a transgenic model of reduced myofibrillar Ca^2+^-sensitivity, SERCA2 deficiency caused no enhancement of the disease state. However, when combined with a transgenic model of increased myofibrillar Ca^2+^-sensitivity, SERCA2 haploinsufficiency caused rapid onset of hypertrophy, decompensation, and death. These effects were associated with reduced expression of the antiapoptotic *Hax1*, increased levels of the proapoptotic genes *Chop* and *Casp12*, and evidence of perturbations in energy metabolism. These data reveal myofibrillar Ca^2+^-sensitivity to be an important determinant of the cardiac effects of SERCA2 haploinsufficiency and raise the possibility that Darier disease patients are more susceptible to heart failure under certain conditions.

## 1. Introduction

In humans, loss of one copy of the* ATP2A2* gene causes Darier disease (DD), an acantholytic skin disease [1, 2].* ATP2A2* encodes two alternatively spliced variants of sarco/endoplasmic reticulum Ca^2+^-ATPase isoform 2 (SERCA2). These are SERCA2b, the ubiquitous endoplasmic reticulum (ER) Ca^2+^ pump, and SERCA2a, the cardiac and slow-twitch skeletal muscle sarcoplasmic reticulum (SR) Ca^2+^ pump [3]. In its capacity as the primary cardiac SR Ca^2+^ pump, SERCA2a facilitates muscle relaxation and replenishes SR Ca^2+^ stores needed for contraction [3]. Because reduced SERCA2 activity is often observed in heart disease [4] and DD mutations cause reductions in SERCA2 expression and activity [5], one might expect these mutations to lead to heart disease in humans. However, two studies of DD patients with a mean age of ~47 years indicated that cardiac performance is normal and yielded no evidence of heart disease [6, 7]; these findings have been interpreted to suggest that a role for SERCA2 deficiency in heart disease, while relevant in rodent models, may be less important in humans [7]. However, while these results provide compelling evidence that* Atp2a2* heterozygosity does not impair cardiac function in middle-aged humans, they do not rule out the possibility that SERCA2 haploinsufficiency can increase susceptibility to disease progression and heart failure.

In earlier studies, ablation of one copy of the* Atp2a2* gene in mice of a mixed 129Svj and Black Swiss background caused a reduction in cardiac SERCA2a protein expression to 65% of wild-type (WT) levels, with impaired contractility and relaxation [8]. However, true heart disease was not observed; in fact, the major phenotype of the* Atp2a2* ^+/−^ mice was the development of squamous cell tumors in keratinized epithelial tissues [9, 10]. Later studies on these mice revealed an increased susceptibility to pressure-overload cardiac hypertrophy [11] and a reduction of rate-dependent inotropy in isolated mutant hearts, relative to WT controls [12]. In the current study, we used* Atp2a2* ^+/−^ mice on an inbred FVB/N background; while these heterozygous (HET) mice continued to display effects of SERCA2 haploinsufficiency in keratinized epithelia, cardiac performance was apparently normal, more closely reflecting findings in DD patients [6, 7]. The HET model was well suited to determine if SERCA2 deficiency, while apparently benign under normal conditions, could exacerbate cardiac disease progression. Specifically, we investigated the interaction of SERCA2 haploinsufficiency with hypothyroidism, which is known to impair cardiac performance [13], and alterations in myofibrillar Ca^2+^-sensitivity, which causes pathological hypertrophy and heart failure [14, 15]. For the latter, double mutant mice were generated by crossing HET mice with transgenic lines expressing the Glu54Lys mutant *α*-tropomyosin, which reduces myofibrillar Ca^2+^-sensitivity and leads to dilated cardiomyopathy [16, 17], and the Glu180Gly mutant *α*-tropomyosin, which increases myofibrillar Ca^2+^ sensitivity and causes hypertrophic cardiomyopathy [18, 19]. Our results reveal an unexpected selectivity in the effects of SERCA2 haploinsufficiency in heart, which should be taken into consideration in the management of Darier disease patients.

## 2. Materials and Methods

### 2.1. Animal Models

The original* Atp2a2* ^+/−^ line was backcrossed onto the inbred FVB/N background in excess of 15 generations to generate the HET mutant line utilized in this study. This* Atp2a2* ^+/−^ mouse line has been made available to researchers through the Jackson Labs repository. All other mice used in this study, including the WT, the transgenic mouse models carrying the Glu154Lys mutant *α*-tropomyosin, which has dilated cardiomyopathy (DCM) [16], and the Glu180Gly mutant *α*-tropomyosin, which has hypertrophic cardiomyopathy (HCM) [18], were also on the inbred FVB/N background. HET mice were bred with the DCM and HCM lines to generate double (DCM/HET and HCM/HET) and single mutant offspring. The hypothyroid model was prepared by treating WT and HET mice with 6-n-propyl thiouracil (PTU) exactly as previously described [20]. All procedures conformed to guidelines published by the NIH (*Guide for the Care and Use of Laboratory Animals*; publication number 86-23, revised 1996) and were approved by the Institutional Animal Care and Use Committee at the University of Cincinnati.

### 2.2. Evaluation of Cardiac Function

Anesthesia of mice with ketamine and inactin, analysis of cardiovascular function using pressure transducers inserted into the left ventricle and right femoral vein, delivery of drugs* via* a cannula in the right femoral vein, and recording and analysis of data were performed exactly as described previously [13]. Analysis of cardiac function by M-mode echocardiography of mice, anesthetized using isoflurane inhalation, and analysis of data were performed exactly as described previously [21].

### 2.3. Immunoblot Analyses

Hearts were harvested from anesthetized mice and processed for immunoblot analysis as previously described [22]. Phosphorylation of phospholamban (PLN) in response to *β*-adrenergic stimulation was assessed in ventricles from mice that were anesthetized and surgically instrumented as described above and treated with dobutamine (16 ng/g body weight/min). Estimation of protein concentration in total homogenates, resolution of proteins by discontinuous, reducing SDS-PAGE, and immunoblot analyses were carried out as described [22]. All primary and secondary antibodies used have been previously described [22].

### 2.4. Real-Time Polymerase Chain Reaction

Hearts/ventricles were harvested from anesthetized mice and processed for real-time PCR (RT-PCR) analysis as previously described [22]. In addition to the primer pairs that have been previously described [22, 23], the following were used:* Hspa5* (GRP78/BiP), PrimerBank ID number 31981722a1;* Hsp90b1* (GRP94), PrimerBank ID number 6755863a1;* Casp12* (Caspase 12), PrimerBank ID number 31981868a1;* Ddit3* (CHOP), PrimerBank ID number 31982415a1;* Eif2ak3* (PERK), PrimerBank ID number 6857781a1;* Acox1*, PrimerBank ID number 26333821a1;* Fabp3*, PrimerBank ID number 6753810a1;* Orai1*, PrimerBank ID number 93277106b1;* Stim1*, PrimerBank ID number 31981983a2;* Hax1*, PrimerBank ID number 6754160a1;* Rcan2*, PrimerBank ID number 46560586c1; and* Pparγ*, PrimerBank ID number 187960104c1; primers for* Pln* (phospholamban) were adapted from PrimerBank ID number 213512815c1 (forward primer: 5′ AAGTGCAATACCTCACTCG 3′, reverse primer: 5′ GATCAGCAGCAGACATATC 3′). mRNA levels for* Atp2b1* (QT01072106) and* Atp2b4* (QT01076271) were determined using QuantiTect Primer Assay Kits (Qiagen).

### 2.5. Statistics

Results are presented as means ± standard error (SE). Individual comparisons were performed using a two-sided Student's *t*-test, and a *P* value of <0.05 was considered significant.

## 3. Results

### 3.1. Cardiovascular Performance in WT and HET Mice

**Figure 1:**
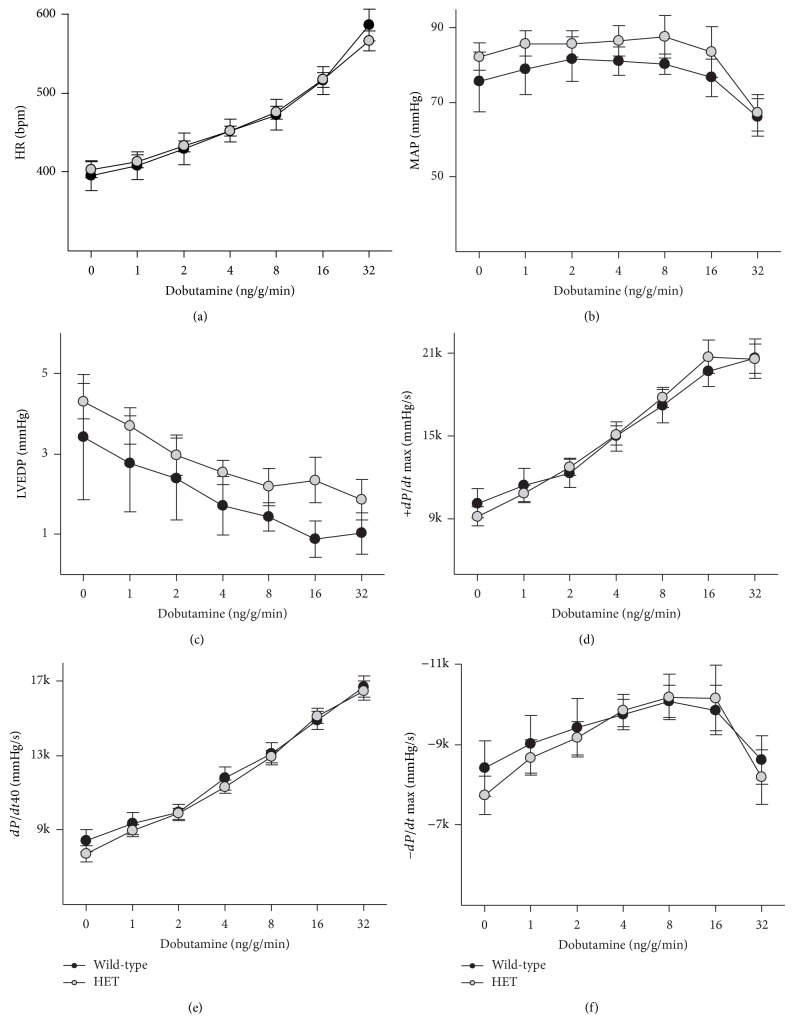
Cardiovascular function in* Atp2a2* heterozygous mice. Ventricular and arterial pressures were measured in anesthetized adult FVB/N wild-type and* Atp2a2* ^+/−^ mice under baseline conditions and upon *β*-adrenergic stimulation using dobutamine. (a) Heart rate (HR); (b) mean arterial pressure (MAP); (c) left ventricular end-diastolic pressure (LVEDP); (d) maximal rate of left ventricular pressure development (+dP/dt Max); (e) rate of left ventricular pressure development at 40 mmHg (dP/dt40); and (f) maximal rate of decay of left ventricular developed pressure (−dP/dt Max). Values are means ± SE. *n* = at least 5 for each genotype.

Cardiovascular performance of adult FVB/N WT and HET mice was analyzed using a pressure transducer in the left ventricle under both basal conditions and upon *β*-adrenergic stimulation with dobutamine. No significant differences were observed in basal heart rate, mean arterial pressure, left ventricular end-diastolic pressure, or maximum rates of left ventricular pressure development or decay (Figure 1). Treatment with dobutamine led to similar changes in both genotypes, with no impairment of chronotropic, inotropic, or lusitropic responses. Rate of left ventricular pressure development was also calculated at 40 mm Hg, to assess possible effects of the small but nonsignificant difference in mean arterial pressure (Figure 1(b)); however the two genotypes had virtually identical responses.

### 3.2. Effects of* Atp2a2* Heterozygosity on Proteins Implicated in Ca^2+^-Handling

**Figure 2:**
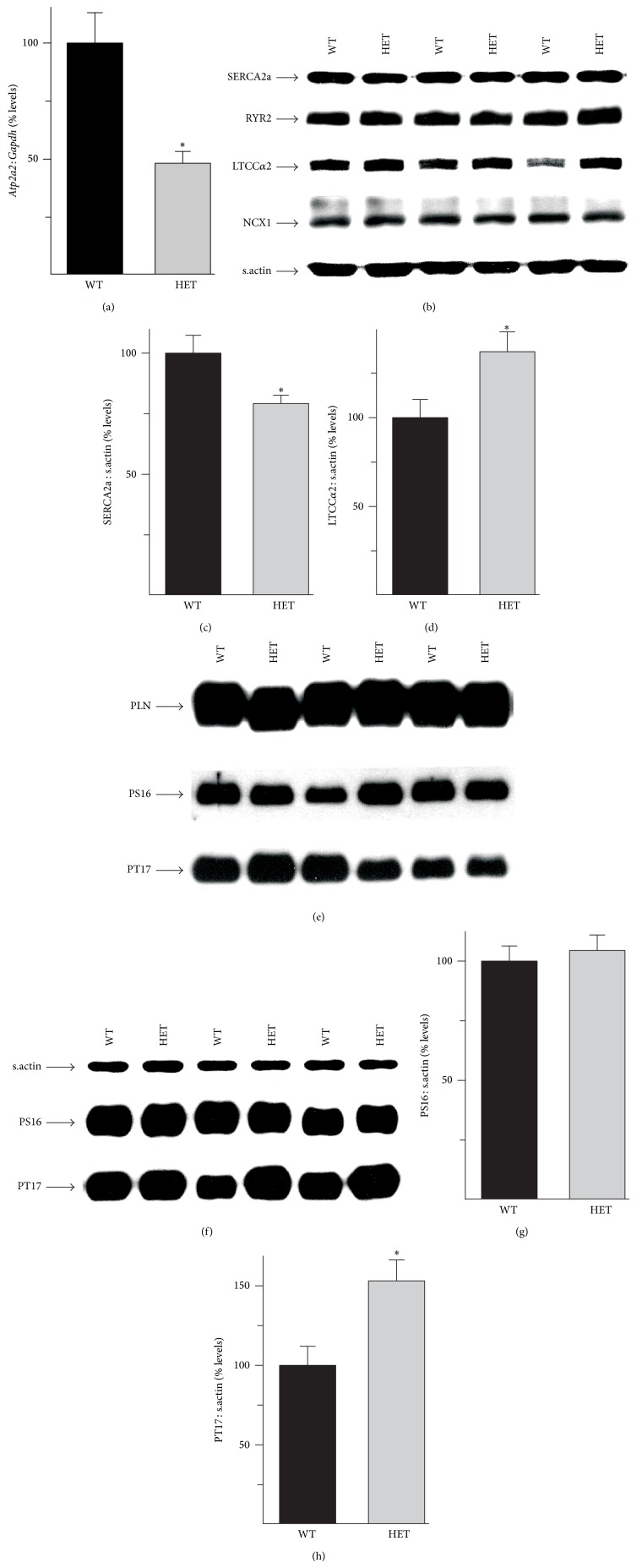
Effects of* Atp2a2* heterozygosity on expression of Ca^2+^ handling proteins in heart. Adult WT and* Atp2a2* ^+/−^ (HET) hearts were processed for analysis of mRNA and protein levels. (a)* Atp2a2* mRNA determined by RT-PCR; (b) immunoblot analysis of SERCA2a, ryanodine receptor isoform 2 (RYR2), *α*2 subunit of L-type Ca^2+^ channel (LTCC*α*2), and Na^+^/Ca^2+^ exchanger isoform 1 (NCX1). Quantitation of SERCA2a (c) and LTCC*α*2 (d) protein levels. Immunoblot analyses of phospholamban (PLN) and PLN phosphorylated on Ser16 (PS16) and Thr17 (PT17) were performed using heart samples from anesthetized surgically instrumented mice under both baseline conditions (e) and after *β*-adrenergic stimulation with dobutamine at 16 ng/g body weight/min (f, g, h). mRNA levels were normalized to* Gapdh* and protein levels were normalized to sarcomeric actin (s.actin). Values are means ± SE. *n* = at least 4 for each genotype. ^∗^ *P* < 0.05 versus WT controls.

RT-PCR analysis of total RNA from WT and HET hearts revealed that mRNA levels of the* Atp2a2* gene, which codes for SERCA2, were reduced to 48 ± 5% of WT levels (Figure 2(a)). However, immunoblot analysis of total protein homogenates revealed a much smaller reduction in SERCA2a protein levels (to 79 ± 3% of WT levels, Figures 2(b) and 2(c)) in HET hearts. This reduction was associated with an increase (to 137 ± 11% of WT levels, Figures 2(b) and 2(d)) in expression of the *α*2 subunit of the L-type Ca^2+^-channel, which is the principal Ca^2+^-uptake mechanism on a beat-to-beat basis. There were no changes in expression of either the ryanodine receptor isoform 2, which mediates Ca^2+^-release from the SR, or the Na^+^/Ca^2+^ exchanger isoform 1 (NCX1,* Slc8a1*), which is the predominant Ca^2+^-efflux mechanism in cardiac myocytes, in HET hearts (Figure 2(b)).

SERCA2 activity is negatively regulated by phospholamban (PLN), which binds to the Ca^2+^-pump in a phosphorylation-dependent manner [24]. Reduced PLN expression and increased PLN phosphorylation on residues Ser16 (PS16) and Thr17 (PT17) can both enhance SERCA2a-mediated SR Ca^2+^-sequestration [24]. However, immunoblot analysis revealed that PLN expression and baseline phosphorylation (Figure 2(e)) were unaltered in HET hearts. The inotropic and lusitropic effects of *β*-adrenergic stimulation are mediated, at least in part, via increased PLN phosphorylation, which facilitates more robust Ca^2+^-cycling [24]. The normal increase in the cardiovascular performance of stimulated HET mice raised the possibility that *β*-adrenergic stimulated PLN phosphorylation was augmented to compensate for the reduction in SERCA2a levels. To test this hypothesis, PS16 and PT17 levels were determined in hearts from WT and HET mice stimulated with dobutamine. While PS16 levels were comparable between stimulated WT and HET hearts (Figures 2(f) and 2(g)), PT17 levels were elevated in stimulated HET hearts (by 153 ± 13%), when compared to similarly treated WT hearts (Figures 2(f) and 2(h)).

### 3.3. *Atp2a2* Heterozygosity Does Not Exacerbate Cardiac Dysfunction Caused by Hypothyroidism

**Figure 3:**
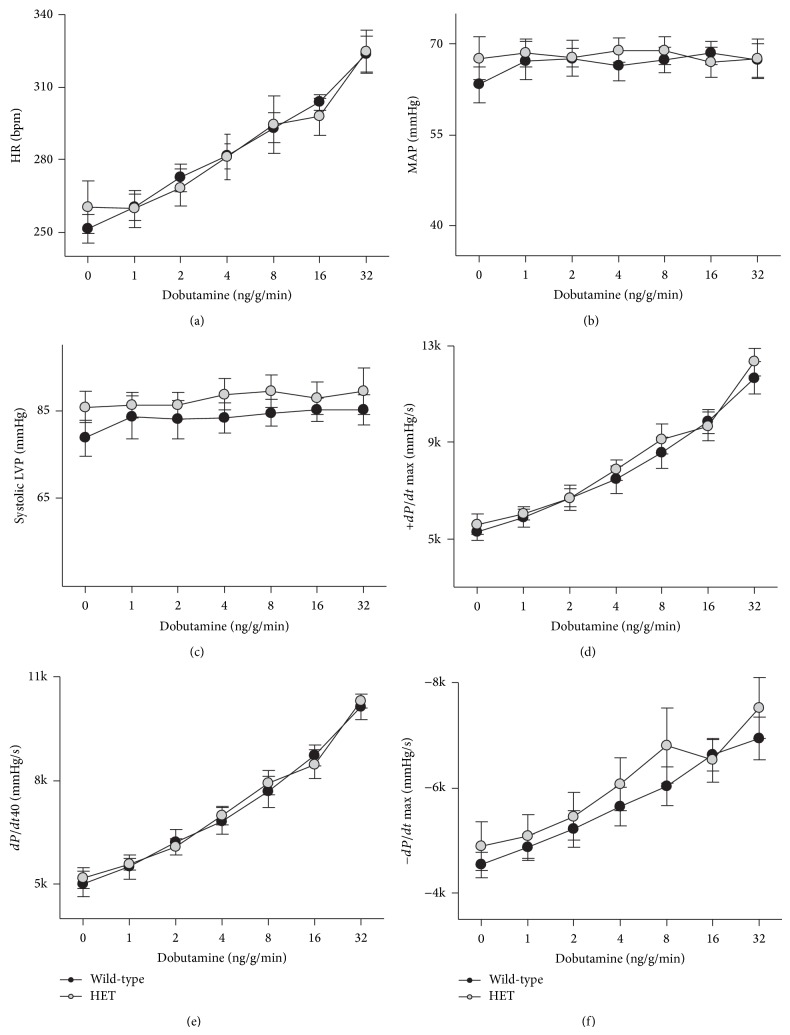
Effects of hypothyroidism on cardiovascular performance of* Atp2a2* heterozygous mice. Adult wild-type and* Atp2a2* ^+/−^ (HET) mice were rendered hypothyroid and cardiovascular performance was assessed under both baseline conditions and after *β*-adrenergic stimulation. (a) Heart rate (HR); (b) mean arterial pressure (MAP); (c) systolic left ventricular pressure (systolic LVP); (d) maximal rate of left ventricular pressure development (+dP/dt Max); (e) rate of left ventricular pressure development at 40 mmHg (dP/dt40); (f) maximal rate of decay of left ventricular developed pressure (−dP/dt Max). Values are means ± SE. *n* = at least 4 for each genotype.

Hypothyroidism is a well-recognized cardiovascular disease risk factor [25]. Studies in rabbits and mice have shown that it is associated with a reduction in cardiac SERCA2 expression [20, 26], raising the possibility that the impairment of cardiac function caused by hypothyroidism would be greater in* Atp2a2* heterozygous individuals. To test this hypothesis, hypothyroidism was induced in WT and HET mice and cardiac performance was assessed by* in vivo* catheterization. Cardiovascular function was similarly diminished in both WT and HET mice (Figures 3(a)–3(f)) under both baseline conditions and upon *β*-adrenergic stimulation.

### 3.4. *Atp2a2* Heterozygosity Has No Appreciable Effect on the Cardiac Phenotype of Transgenic Mice with Dilated Cardiomyopathy

**Figure 4:**
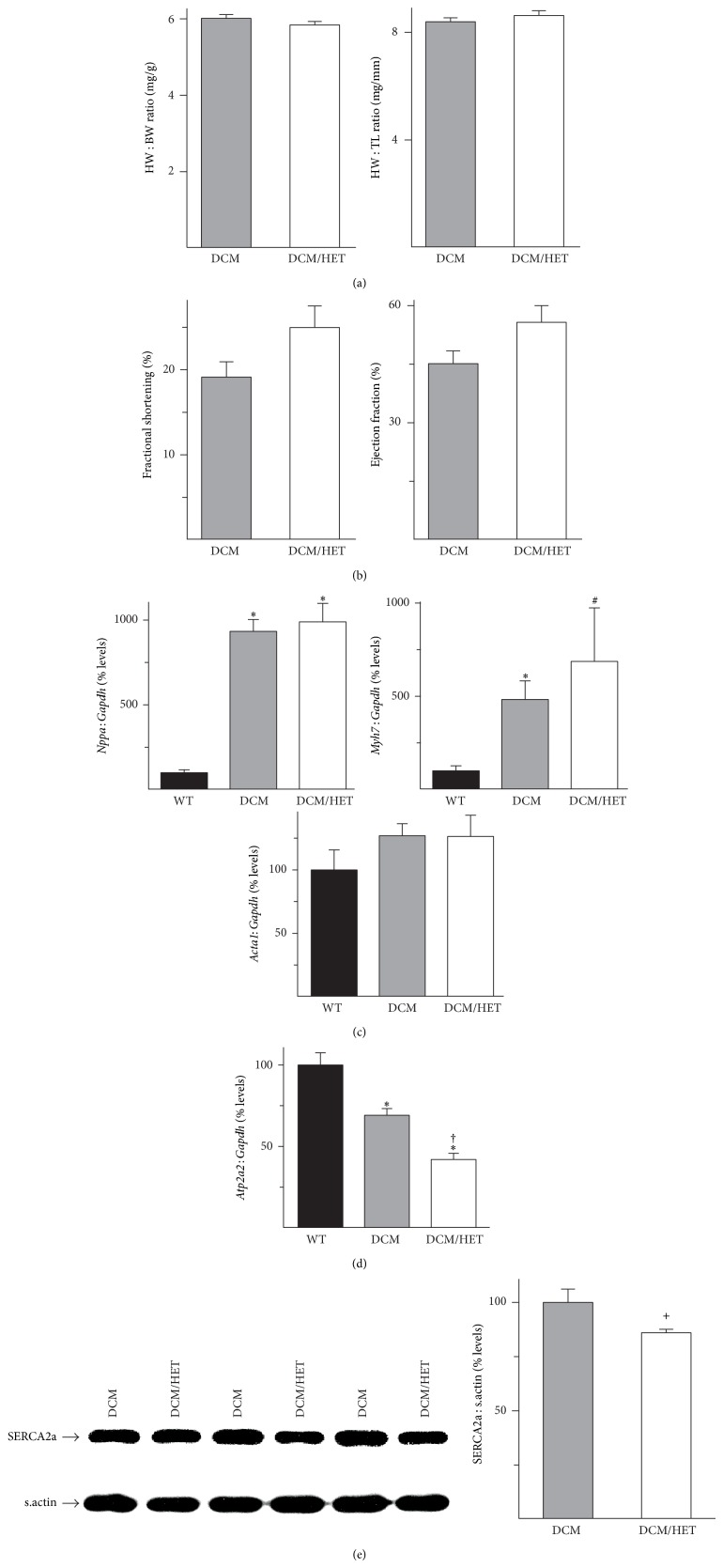
Effects of* Atp2a2* heterozygosity in a transgenic model of reduced myofibrillar Ca^2+^ sensitivity. WT mice, transgenic mice expressing the Glu154Lys mutant *α*-tropomyosin, which causes dilated cardiomyopathy (DCM), and double mutant DCM/*Atp2a2* ^+/−^ (DCM/HET) mice were analyzed. Morphometric analyses revealed similar heart weight : body weight (HW : BW) and heart weight : tibial length (HW : TL) ratios in DCM and DCM/HET mice (a); echocardiographic analysis shows fractional shortening and ejection fraction in DCM and DCM/HET mice (b); RT-PCR analysis shows mRNA levels for (c) atrial natriuretic peptide (*Nppa*), *β*-myosin heavy chain (*Myh7*), and skeletal *α*-actin (*Acta1*) and for* Atp2a2* (d). Immunoblot analysis of cardiac homogenates and quantitation show relative levels of SERCA2a (e) in DCM and DCM/HET hearts. mRNA levels were normalized to* Gapdh* and protein levels were normalized to sarcomeric actin (s.actin). Values are means ± SE. *n* = at least 4 for each genotype. ^∗^ *P* < 0.05 versus WT controls; ^#^ *P* = 0.06 versus WT controls; ^+^ *P* = 0.05 versus DCM; ^†^ *P* < 0.05 versus DCM.

Myofibrillar Ca^2+^-sensitivity is a major determinant of cardiac function; in addition to altering force development, changes in Ca^2+^-sensitivity can impact myofibrillar Ca^2+^-buffering, diastolic Ca^2+^-levels, and SERCA2-mediated cytosolic Ca^2+^-clearance [14, 15, 27–29]. Transgenic expression of Glu154Lys mutant *α*-tropomyosin in the DCM mouse heart lowers myofibrillar Ca^2+^ sensitivity, impairs contractility, and leads to dilated cardiomyopathy [16]. In order to determine the effects of SERCA2 haploinsufficiency, double mutant DCM/HET mice were analyzed. DCM/HET mice were viable and appeared normal. The increase in heart weight : body weight (HW : BW) and heart weight : tibial length ratios, determined in 8–10-week-old mice, was similar in both single mutant DCM and double mutant DCM/HET mice (Figure 4(a)). Echocardiographic analyses revealed that cardiac function was not compromised in DCM/HET mice when compared to DCM controls; in fact, fractional shortening and ejection fraction trended slightly higher in DCM/HET hearts (Figure 4(b)). RT-PCR analysis showed that the increases in mRNA levels for* Nppa* (atrial natriuretic peptide),* Myh7* (*β*-myosin heavy chain), and* Acta1* (skeletal *α*-actin), as markers of pathological hypertrophy, were not significantly different between DCM and DCM/HET hearts (Figure 4(c)).* Atp2a2* mRNA levels, which were reduced to 69 ± 4% of WT levels in DCM hearts, were lower in DCM/HET hearts (42 ± 4% of WT levels; Figure 4(d)). However, the reduction in SERCA2a protein was more modest, with expression at 86 ± 2% of DCM levels in DCM/HET hearts (Figure 4(e)).

### 3.5. *Atp2a2* Heterozygosity Causes Rapid Progression of Hypertrophy and Decompensation in Transgenic HCM Mice

**Figure 5:**
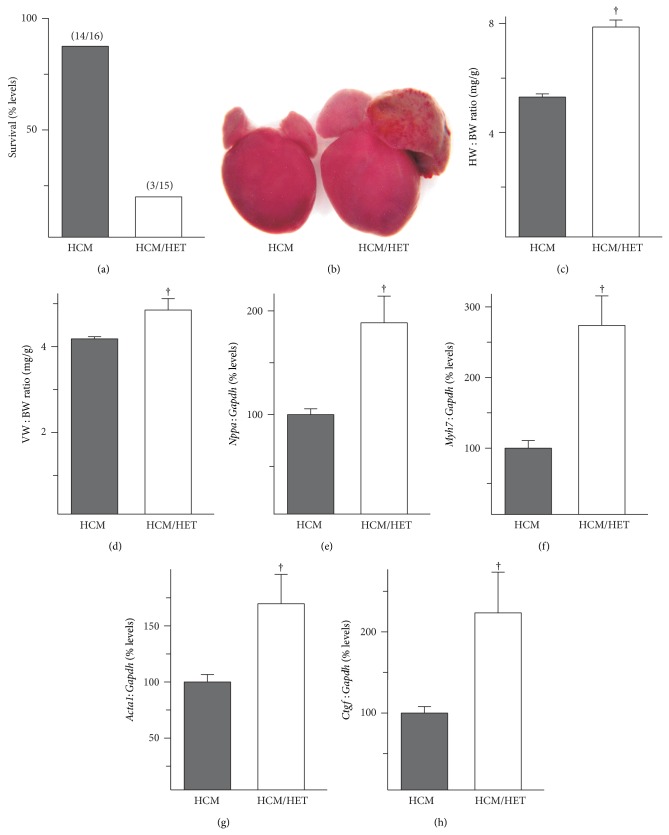
Effects of* Atp2a2* heterozygosity in a transgenic model of increased myofibrillar Ca^2+^ sensitivity. WT mice, mice expressing the Glu180Gly mutant *α*-tropomyosin, which causes hypertrophic cardiomyopathy (HCM), and double mutant HCM/*Atp2a2* ^+/−^ (HCM/HET) mice were analyzed. Survival of HCM and HCM/HET mice was assessed at 5 weeks of age (a). Gross morphometry at 4 weeks of age showed (b) overt remodeling, (c) increased heart weight : body weight ratios (HW : BW), and (d) increased ventricular weight : body weight ratios (VW : BW) in HCM/HET mice. RT-PCR shows elevated mRNA levels in HCM/HET hearts for (e) atrial natriuretic peptide (*Nppa*), (f) *β*-myosin heavy chain (*Myh7*), (g) skeletal *α*-actin (*Acta1*), and (h) connective tissue growth factor (*Ctgf*). mRNA levels were normalized to* Gapdh* expression. Values shown are means ± SE. *n* = at least 4 for each genotype. ^†^ *P* < 0.05 versus HCM controls.

Increased myofibrillar Ca^2+^-sensitivity, which impairs relaxation, is associated with hypertrophic cardiomyopathy and has also been reported in end-stage heart failure [14, 15]. Cardiac expression of Glu180Gly mutant *α*-tropomyosin in the HCM transgenic model increases myofibrillar Ca^2+^-sensitivity, impairs relaxation, leads to development of fibrosis and hypertrophy, and causes death at 5-6 months of age [18]. To determine the effects of SERCA2 haploinsufficiency on the HCM phenotype, double mutant HCM/HET mice were generated as described above. SERCA2 haploinsufficiency caused a rapid onset of heart failure and death; 80% of HCM/HET mice were dead by 5 weeks of age and none survived beyond 6-7 weeks (Figure 5(a)). When compared to age-matched HCM controls, HCM/HET hearts displayed overt hypertrophy with pronounced left atrial remodeling as early as 4 weeks of age (Figure 5(b)). HW : BW ratios were higher in HCM/HET mice (Figure 5(c)), as were ratios of ventricular weight : body weight (Figure 5(d)), indicating that the increase in HW : BW was not simply a consequence of atrial remodeling. Consistent with these changes, mRNA levels for several markers of cardiac remodeling, which have been shown to be elevated in 4-week-old HCM hearts [23], were further increased in HCM/HET hearts; these included* Nppa* (Figure 5(e)),* Myh7* (Figure 5(f)),* Acta1* (Figure 5(g)), and* Ctgf*, encoding connective tissue growth factor (Figure 5(h)).

### 3.6. Effect of* Atp2a2* Heterozygosity on Regulators of Ca^2+^-Handling and Protein Phosphatases in HCM/HET Hearts

**Figure 6:**
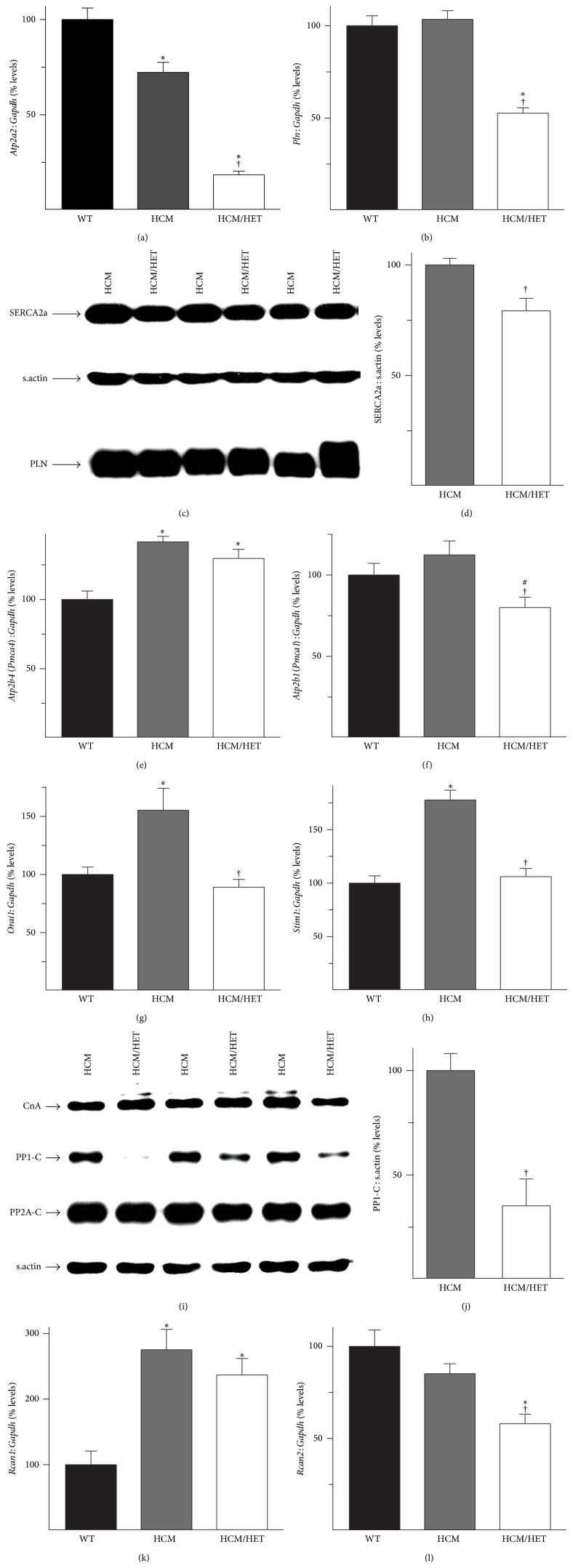
Effects of* Atp2a2* heterozygosity on regulators of Ca^2+^-handling and phosphatases in HCM models. Hearts from WT mice, mice expressing the Glu180Gly mutant *α*-tropomyosin (HCM), and HCM/*Atp2a2* ^+/−^ double mutant (HCM/HET) mice were processed for RT-PCR and immunoblots. RT-PCR analysis of mRNA for (a) SERCA2 (*Atp2a2*) and (b) phospholamban (*Pln*), (c) immunoblot analysis of SERCA2a and PLN, and (d) quantitation of SERCA2a protein. RT-PCR analyses of mRNA for (e) plasma membrane Ca^2+^-ATPase isoform 4 (*Atp2b4*), (f) plasma membrane Ca^2+^-ATPase isoform 1 (*Atp2b1*), (g) sarcolemmal calcium release-activated calcium modulator 1 (*Orai1*), and (h) stromal interaction molecule 1 (*Stim1*) and (i) immunoblot analyses of the catalytic subunits of calcineurin (CnA), protein phosphatase 1, (PP1-C), and protein phosphatase 2A (PP2A-C) in HCM and HCM/HET hearts. (j) Quantitation of PP1-C protein levels. RT-PCR analyses of mRNA for (k) regulator of calcineurin 1 (*Rcan1*) and (l) regulator of calcineurin 2 (*Rcan2*). mRNA levels were normalized to* Gapdh* and protein levels were normalized to sarcomeric actin (s.actin). Values are means ± SE. *n* = at least 4 for each genotype. ^∗^ *P* < 0.05 versus WT controls; ^†^ *P* < 0.05 versus HCM; ^#^ *P* = 0.08 versus WT controls.

RT-PCR analysis of 4-week-old WT, HCM, and HCM/HET hearts revealed that* Atp2a2* mRNA levels, which were reduced to 72 ± 5% of WT levels in HCM hearts, were sharply reduced in HCM/HET hearts (to 18 ± 2% of WT levels; Figure 6(a)). mRNA levels for PLN, which were maintained at WT levels in HCM hearts, were also reduced in HCM/HET hearts (53 ± 3% of WT levels; Figure 6(b)). However, expression of SERCA2a protein showed a smaller reduction of just 23 ± 5% from HCM levels in HCM/HET hearts (Figures 6(c) and 6(d)), with no change seen in PLN protein levels (Figure 6(c)).

In addition to its role in contractility, Ca^2+^ is a regulator of key signaling cascades in heart; the Ca^2+^-pools implicated in such regulatory functions are thought to exist in microdomains spatially distinct from bulk SR Ca^2+^-stores [30], which are probably regulated by sarcolemmal Ca^2+^ pumps such as the plasma membrane Ca^2+^-ATPases (PMCA). Impairments in cytosolic bulk Ca^2+^-handling have the potential to perturb the spatial isolation of these Ca^2+^-microdomains and can lead to a greater reliance on sarcolemmal Ca^2+^ pumps for Ca^2+^-clearance. RT-PCR analysis revealed that mRNA levels for PMCA4 were increased in HCM hearts (141 ± 4% of WT levels) and remained similarly elevated in HCM/HET hearts (Figure 6(e)). In contrast, mRNA levels for PMCA1, which were not significantly altered in HCM hearts, were reduced in HCM/HET hearts (to 80 ± 6% of WT levels, Figure 6(f)).

As described above, increased myofibrillar Ca^2+^-buffering in HCM hearts has the potential to antagonize SR Ca^2+^-sequestration, with effects on SR/ER Ca^2+^-store levels. However, as we have previously demonstrated [22], the amplitude of stimulated Ca^2+^-transients is only modestly reduced in HCM myocytes, which raises the possibility that SR/ER Ca^2+^-stores may be replenished by alternative mechanisms in these hearts. These can include mediators of store-operated Ca^2+^-entry (SOCE); indeed, expression of mRNAs for both* Orai1* (to 155 ± 19% of WT levels) and* Stim1* (to 178 ± 9% of WT levels), which encode proteins with well-characterized roles in stress-induced SOCE activity [31, 32], was increased in HCM hearts. Unexpectedly, this increase was reversed to WT levels in HCM/HET hearts (Figures 6(g) and 6(h)).

Increased expression or activity of the protein phosphatases calcineurin, protein phosphatase 1 (PP1), and protein phosphatase 2A (PP2A) is strongly associated with pathological hypertrophy and heart failure [33–36]. Protein levels of the catalytic subunits of calcineurin (CnA), PP1 (PP1-C), and PP2A (PP2A-C), which reflect expression of the respective holoenzymes, were assessed. While CnA and PP2A-C expression were comparable between HCM and HCM/HET hearts (Figure 6(i)), PP1-C levels were reduced in HCM/HET hearts (Figures 6(i) and 6(j)). To address the possibility that calcineurin activity was augmented in HCM/HET hearts, mRNA levels for regulator of calcineurin 1 (calcipressin-1,* Rcan1*), a marker of calcineurin activity, were determined by RT-PCR analysis. While there was no difference in* Rcan1* levels between HCM and HCM/HET hearts (Figure 6(k)), mRNA levels of* Rcan2*, which codes for calcipressin-2, were significantly reduced in HCM/HET hearts (58 ± 5% of WT levels; Figure 6(l)).

### 3.7. Expression of ER Stress Markers and Regulators of Apoptosis in HCM/HET Hearts

**Figure 7:**
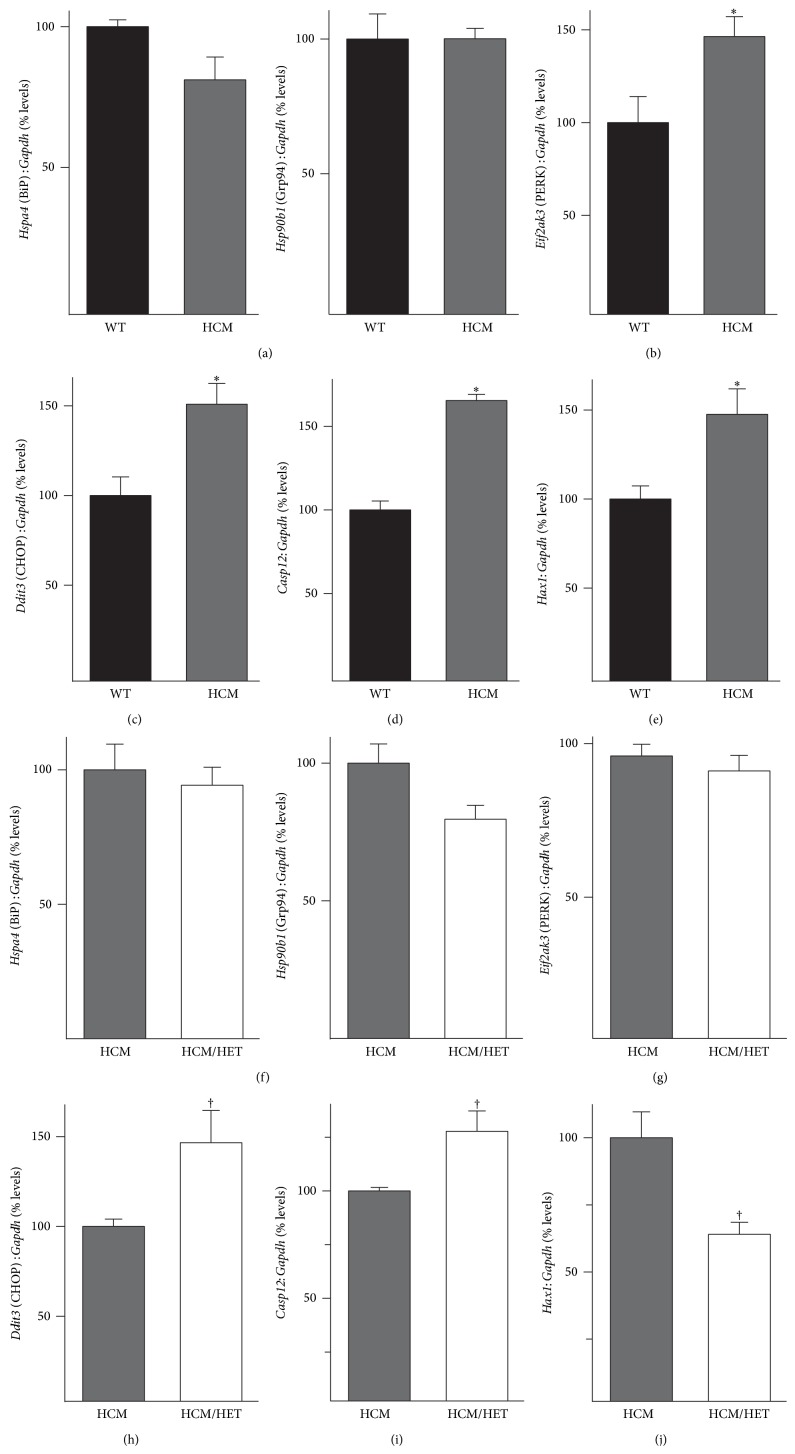
Effect of* Atp2a2* heterozygosity on markers of ER stress and apoptosis in HCM and HCM/HET hearts. Heart RNA from WT mice, mice expressing the Glu180Gly mutant *α*-tropomyosin (HCM), and HCM/*Atp2a2* ^+/−^ double mutant (HCM/HET) mice was analyzed by RT-PCR. Panels (a)–(e) compare WT and HCM; panels (f)–(j) compare HCM and HCM/HET. mRNA levels are shown for (a, f) ER chaperones BiP/GRP78 (*Hspa4*) and GRP94 (*Hsp90b1*); (b, g) PERK (*Eif2ak3*); (c, h) CHOP (*Ddit3*); (d, i) caspase 12 (*Casp12*); and (e, j) HCLS1 associated protein X-1 (*Hax1*) in WT, HCM, and HCM/HET hearts. mRNA levels were normalized to* Gapdh*. Values shown are means ± SE. *n* = at least 4 for each genotype. ^∗^ *P* < 0.05 versus WT controls; ^†^ *P* < 0.05 versus HCM.

The reduction in SERCA2a protein levels, coupled with the downregulation of SOCE-related genes, raised the possibility that ER stress was elevated in HCM/HET hearts. We initially assessed expression of key ER stress markers in HCM hearts. RT-PCR analysis revealed no increase in mRNA for the ER chaperones BiP/GRP78 or GRP94 (Figure 7(a)). However, mRNA for PERK, a major kinase involved in ER stress responses, was increased in HCM hearts (to 147 ± 12% of WT levels; Figure 7(b)). Furthermore, mRNAs for the ER stress related proapoptotic proteins CHOP (145 ± 12% of WT levels; Figure 7(c)) and CASP12 (164 ± 5% of WT levels; Figure 7(d)) were also increased in HCM hearts. These changes were associated with an increase (to 148 ± 14% of WT levels; Figure 7(e)) in mRNA for the antiapoptotic protein HCLS1 associated protein X-1 (HAX1), which is localized to mitochondria and the SR [37].

There was no increase in mRNA levels for GRP78, GRP94, or PERK in HCM/HET hearts when compared to HCM controls (Figures 7(f) and 7(g)). However, mRNA levels for CHOP (143 ± 16% of HCM levels; Figure 7(h)) and CASP12 (128 ± 9% of HCM levels; Figure 7(i)) were further elevated in HCM/HET hearts. The increase seen in HAX1 mRNA in HCM hearts was reversed in HCM/HET hearts (to 64 ± 4% of HCM levels; Figure 7(j)).

### 3.8. Effect of* Atp2a2* Heterozygosity on Regulators of Energy Metabolism in HCM Hearts

**Figure 8:**
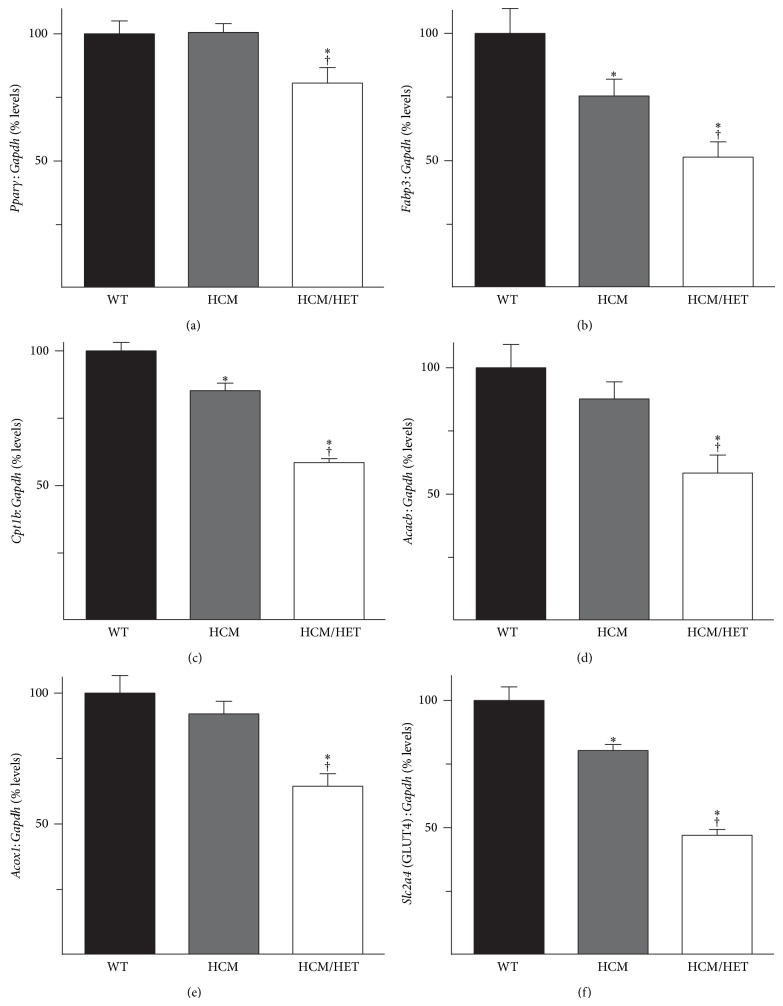
Effect of* Atp2a2* heterozygosity on regulators of energy metabolism in HCM and HCM/HET hearts. Heart RNA from WT mice, mice expressing the Glu180Gly mutant *α*-tropomyosin (HCM), and HCM/*Atp2a2* ^+/−^ double mutant (HCM/HET) mice was analyzed by RT-PCR. mRNA levels are shown for (a) peroxisome proliferator-activated receptor gamma (*Pparγ*); (b) fatty acid binding protein 3 (*Fabp3*); (c) the muscle-isoform of carnitine palmitoyltransferase 1 (*Cpt1b*); (d) acetyl CoA-carboxylase beta (*Acacb*); (e) acyl-CoA oxidase 1 (*Acox1*); and (f) the GLUT4 glucose transporter (*Slc2a4*). mRNA levels were normalized to* Gapdh*. Values are means ± SE. *n* = at least 4 for each genotype. ^∗^ *P* < 0.05 versus WT controls; ^†^ *P* < 0.05 versus HCM.

Impaired relaxation resulting from an increase in myofibrillar Ca^2+^-sensitivity can energetically stress the heart [38]. The resultant dysregulation of energy metabolism has been suggested to contribute to the hypertrophic phenotype associated with sarcomeric mutations that increase myofibrillar Ca^2+^-sensitivity [39, 40]. mRNA levels of* Pparγ*, encoding peroxisome proliferator-activated receptor gamma, a regulator of lipid metabolism that is implicated in the development of pathological hypertrophy [41], were normal in HCM hearts but reduced in HCM/HET hearts (to 81 ± 6% of WT levels; Figure 8(a)). Hearts rely predominantly on lipids for their energy supply [42] and FABP3 is the cardiac/muscle-isoform of fatty acid binding proteins, which mediate the intracellular transport of long-chain fatty acids. Expression of the* Fabp3* gene was downregulated (to 76 ± 9% of WT levels) in HCM hearts and further reduced in HCM/HET hearts (to 49 ± 5% of WT levels; Figure 8(b)). Mitochondrial uptake of long-chain fatty acids is mediated by carnitine palmitoyltransferase 1b (CPT1b), the mRNA levels for which were reduced in HCM (to 88 ± 3% of WT levels) and further reduced in HCM/HET (to 61 ± 2% of WT levels) hearts (Figure 8(c)). CPT1b-mediated transfer of long-chain fatty acids can be inhibited by malonyl CoA, which is generated from acetyl-CoA by acetyl-CoA carboxylase beta (encoded by* Acacb*). While* Acacb* mRNA levels trended lower in HCM hearts, they were downregulated in HCM/HET hearts (to 58 ± 7% of WT levels; Figure 8(d)). In addition, mRNA levels for acyl-CoA oxidase 1 (*Acox1*), which is the first enzyme of the *β*-oxidation pathway, were also reduced (to 64 ± 5% of WT levels) in HCM/HET hearts (Figure 8(e)). Besides lipids, hearts also utilize glucose as an energy source [42]. Glucose uptake in heart is mediated by members of* Slc2a* glucose transporter family, of which GLUT4 (*Slc2a4*) is the predominant isoform in cardiac myocytes. mRNA levels for GLUT4 were reduced (to 80 ± 2% of WT levels) in HCM hearts, with a more pronounced reduction (to 47 ± 2% of WT levels) in HCM/HET hearts (Figure 8(f)).

### 3.9. Biochemical Effects of* Atp2a2* Heterozygosity Seen in HCM/HET Hearts Are Lacking in DCM/HET Hearts

As described above,* Atp2a2* heterozygosity did not exacerbate the cardiac disease phenotype of DCM mice. Given the dramatic effects of SERCA2 haploinsufficiency in HCM/HET hearts at the RNA/protein levels, we analyzed DCM/HET hearts to identify possible underlying similarities. Several mRNAs analyzed (*Casp12*,* Fabp3*, and* Cpt1b*) were altered in DCM hearts relative to WT hearts. However, RT-PCR analysis revealed that* Atp2a2* heterozygosity did not impact mRNA levels for* Ddit3* (CHOP),* Casp12*, Hax1,* Fabp3*,* Cpt1b*, or* Slc2a4* (GLUT4) in DCM/HET hearts when compared with DCM hearts (Table 1). Immunoblot analyses of total ventricular homogenates of DCM and DCM/HET mice showed no change in protein levels of CnA, PP1-C, or PP2A-C (data not shown).

## 4. Discussion

While there is strong evidence that* ATP2A2* heterozygosity does not impair cardiovascular performance in humans either at rest or during exercise [6, 7], the opportunities to elucidate the molecular mechanisms that allow for cardiac function to be preserved in otherwise healthy DD patients are obviously limited. For example, it remains unknown if loss of one* ATP2A2* allele elicits the compensatory upregulation of the second functional allele or whether SERCA2a haploinsufficiency in human heart leads to a greater, and apparently effective, reliance on transsarcolemmal Ca^2+^-flux. There have also been no studies to determine if DD patients are more susceptible to heart failure and decompensation in the context of secondary pathological insults. The* Atp2a2* heterozygous mouse model on the inbred FVB/N background offered a unique opportunity to address these questions, as it resembles human DD patients in displaying a skin/keratinized epithelial phenotype with no overt effects on cardiovascular function.

Expression of SERCA2a protein levels at ~80% of WT levels in HET hearts demonstrated a remarkable ability to upregulate expression of the single functional* Atp2a2* allele. This was unexpected given the evidence from transgenic lines that it is difficult to appreciably overexpress SERCA2 protein in mouse hearts [43, 44]. Given that* Atp2a2* mRNA levels were reduced by ~50% in HET hearts, it is reasonable to assume that the increase in SERCA2a protein results principally from posttranscriptional adaptations; these could include augmented translation, extended SERCA2 protein half-life, and/or diminished SERCA2 protein degradation. The increase seen in LTCC*α*2 protein levels is consistent with the idea that even modest reductions in SERCA2a expression might lead to a greater reliance on Ca^2+^-handling across the sarcolemma. While such a shift would help preserve Ca^2+^-homeostasis, it is likely to place a greater energetic burden on the heart. SERCA2 is unique in its stoichiometry of transporting 2 Ca^2+^ ions/ATP hydrolyzed. In comparison, transsarcolemmal flux of Ca^2+^ via LTCC-influx, NCX1-efflux (with a stoichiometry of 3Na^+^ : 1Ca^2+^), and Na^+^/K^+^-ATPase-mediated Na^+^ removal effectively doubles the energy cost of Ca^2+^-clearance.

Increased PLN phosphorylation can also serve to compensate for the reduction in SERCA2 levels [23], with evidence of a predominant role for phosphorylation at Ser16, but not Thr17, in *β*-adrenergic stimulation of inotropy and lusitropy in mouse hearts [45]. Therefore, it is difficult to ascribe any part of the normal *β*-adrenergic response seen in HET hearts to increased PLN phosphorylation, given that PS16 levels were unaltered and only PT17 levels were elevated in stimulated HET hearts. While phosphorylation at Thr17 is mediated by Ca^2+^/calmodulin-dependent kinase CaMKII, dephosphorylation is mediated by PP1 [46, 47]. Although immunoblot analysis showed no evidence of a reduction in PP1 expression in HET hearts (data not shown), a clear reduction in PP1 expression was observed in HCM/HET hearts relative to HCM controls, probably providing some compensation for the reduction in SERCA2 protein.

Reduction in SERCA2 expression/activity is closely associated with progression of heart disease and failure. While recent clinical trials provide strong evidence that enhancing SERCA2a expression can alleviate impairment of cardiac function [48–50], it remains unclear whether SERCA2a deficiency can itself precipitate decompensation and heart failure. This is highly relevant to DD patients, particularly in the context of aging or secondary pathological conditions affecting the heart. Hypothyroidism, which affects about 4.6% of the US population [51], is one such condition known to compromise cardiac function [13, 24] and reduce SERCA2 expression [20, 25]. The finding that* Atp2a2* heterozygosity does not exacerbate the effects of hypothyroidism in mice may result from the switch in myosin heavy chain (MHC) isoforms from *α*-MHC to the slower *β*-MHC, which occurs in hypothyroidism, and is known to be energetically favorable [52]. Whether the effects of hypothyroidism are similarly unaffected by SERCA2 haploinsufficiency in human DD patients is an issue that remains to be clarified; it should be evaluated with no preconceptions, as the lack of an effect in the rodent model does not discount possible consequences in human DD patients.

A relative increase in *β*-MHC levels, which commonly occurs in diseased conditions, renders hearts more susceptible to chronic myocardial stress [53]. Although this outcome was not tested in the context of hypothyroidism in HET mice, we investigated the effects of* Atp2a2* heterozygosity in models of altered myofibrillar Ca^2+^-sensitivity, which impose significant chronic contractile stress and are closely associated with the pathogenesis of cardiomyopathies and heart failure [54]. DCM mice, with reduced myofibrillar Ca^2+^-sensitivity, have been reported to develop hypertrophy at 2 months of age, with significant dilation and myocyte disarray occurring by 5 months and mice starting to die at 4–6 months of age [16]. These effects were shown to be associated with downregulation of SERCA2a, consistent with the possibility that* Atp2a2* heterozygosity would hasten disease pathogenesis. The absence of such an effect in DCM/HET mice however revealed that SERCA2 haploinsufficiency has limited effects in the context of reduced myofibrillar Ca^2+^-sensitivity, which occurs during pathological conditions such as inflammation and sepsis [55].

The rapid onset of hypertrophy and decompensation in HCM/HET mice on the other hand strongly suggests that disease pathogenesis associated with increased myofibrillar Ca^2+^-sensitivity may be exacerbated in DD patients. The catastrophic effects of SERCA2 haploinsufficiency in HCM/HET occurred despite SERCA2a protein levels being reduced by just 23% compared to levels in HCM hearts. This indicates that relatively modest reductions in SERCA2a expression can have profound effects in hearts with increased myofibrillar Ca^2+^-sensitivity. This effect is likely due to the fact that, besides factors such as mechanical load and sarcolemmal Ca^2+^-flux, dissociation of Ca^2+^ from myofibrils is facilitated by SERCA2-mediated Ca^2+^-clearance [56]. The importance of this function is expected to be amplified in the context of elevated myofibrillar Ca^2+^-sensitivity, making even small changes in SERCA2 expression/function highly consequential. Recent reports that elevating SERCA2 activity, either by increased expression of SERCA2a or ablation of phospholamban, attenuates disease progression in HCM mice [57, 58] are consistent with this hypothesis.

Perturbations in cytosolic Ca^2+^-clearance are also associated with the generation of ventricular arrhythmias [56], which are strongly implicated in progression to heart failure and sudden death [59, 60]. Targeted SERCA2a gene therapy has been shown, in multiple models, to reduce ventricular arrhythmias in addition to improving cardiac function [4]. The highly advanced disease condition observed in HCM/HET mice as young as 4 weeks of age precluded effective functional analyses of these mice. However, we examined left ventricular pressure measurements in HET mice and found no evidence of extrasystolic beats or other arrhythmias under either basal conditions or upon maximal *β*-adrenergic stimulation (data not shown).

The more rapid decompensation in HCM/HET hearts was associated with a reversal of the increase in* Orai1* and* Stim1* levels seen in HCM hearts. The incidence of increased SOCE via ORAI1 and STIM1 in pathological hypertrophy has implicated these proteins in disease pathogenesis [31]. However, recent data from knockout models reveal a more nuanced role for these proteins in heart, with effects on Ca^2+^-homeostasis and the development of compensatory hypertrophy [61, 62].* Orai1* deficiency was found to exacerbate loss of cardiac function and hasten progression to dilation and was associated with increased apoptosis [63]. Indeed, levels of proapoptotic* Chop* and* Casp12* were elevated in HCM/HET hearts and notably expression of antiapoptotic* Hax1*, which was elevated in HCM hearts, was reduced to WT levels in HCM/HET hearts. There is increasing evidence that the antiapoptotic function of the HAX1 protein is linked to its association with SR Ca^2+^-handling [37, 64], where HAX1 has been shown to localize to the SR in a PLN-dependent manner [65]. HAX1 overexpression, which promotes cell survival, downregulates SERCA2 protein levels, whereas SERCA2 overexpression has been shown to antagonize its protective effects [66, 67]. Therefore, the reversion of HAX1 expression to WT levels may contribute to the preservation of SERCA2 levels in HCM/HET hearts, with potentially maladaptive effects on myocyte cell survival. Further detailed studies will be necessary to fully elucidate possible interactions between HAX1, which has been localized to mitochondria as well [37], and SERCA2 during HCM pathogenesis. Additional perturbations in myocardial energy metabolism, which are strongly implicated in hypertrophy and heart failure [38–41], are also likely to contribute to the rapid decompensation observed in HCM/HET hearts. Reductions in both CPT1b and GLUT4 have been shown to promote pathological hypertrophy and heart failure [68–70]. Conversely, we have recently demonstrated that protection against Tm180-related hypertrophy is associated with preservation of CPT1b and GLUT4 expression at WT levels [23].

In conclusion, our data show that the loss of one copy of the* Atp2a2* gene, which causes reduced expression of SERCA2a in heart, appears to be benign under normal conditions and even in some disease states. This finding is consistent with the results of studies in human DD patients, in which the loss of one* ATP2a2* allele caused no impairment of cardiac performance [6, 7]. However, our studies with the mouse model of DD also reveal that loss of a single copy of* Atp2a2* can lead to much more rapid decompensation, heart failure, and death in mice carrying an HCM mutation that increases myofibrillar Ca^2+^ sensitivity. These results suggest that DD patients may be less tolerant of the changes associated with increased myofibrillar Ca^2+^-sensitivity in heart. In addition to being relevant to pathological conditions such as end-stage heart failure [15], the implications of this finding could extend to the use of therapeutic agents that enhance myofibrillar Ca^2+^-sensitivity in DD patients. For example, although long-term treatment with the Ca^2+^-sensitizer levosimendan improves cardiac function in a model with cardiomyocyte-specific ablation of SERCA2, it also significantly increases fibrosis in SERCA2-deficient hearts [71]. While extrapolation of results from rodent models to human disease merits caution, our results suggest that progression of some types of heart disease is likely to be exacerbated by DD mutations. DD patients and their physicians should therefore be aware of the possibility of adverse interactions between SERCA2 haploinsufficiency and certain pathological conditions affecting cardiovascular health.

## Figures and Tables

**Table 1:** Effect of *Atp2a2* heterozygosity on regulators of apoptosis and energy metabolism in DCM and DCM/HET hearts.

Gene	Relative mRNA levels (normalized to *Gapdh*)		
	WT^¶^	DCM	DCM/HET
CHOP (*Ddit3*)	100 ± 10	103 ± 3	113 ± 10
Caspase 12 (*Casp12*)	100 ± 7	122 ± 3^*^	120 ± 16
HCLS1 associated protein X-1 (*Hax1*)	100 ± 5	91 ± 3	106 ± 12
Fatty acid binding protein 3 (*Fabp3*)	100 ± 7	70 ± 4^*^	75 ± 7^*^
Carnitine palmitoyltransferase 1 (*Cpt1b*)	100 ± 2	77 ± 3^*^	70 ± 8^*^
Glucose transporter 4 (*Slc2a4*)	100 ± 12	88 ± 2	95 ± 8

^¶^Heart RNA from wild-type (WT) mice, transgenic mice expressing Glu154Lys mutant *α*-tropomyosin (DCM), and DCM/*Atp2a2* ^+/−^ double mutant (DCM/HET) mice was analyzed by RT-PCR. Values are mean ± SE; *n* = at least 4 for each genotype; ^*^ *P* < 0.05 versus WT controls.
